# Doping of Sb into Cu_2_ZnSn(S,Se)_4_ absorber layer *via* Se&Sb_2_Se_3_ co-selenization strategy for enhancing open-circuit voltage of kesterite solar cells

**DOI:** 10.3389/fchem.2022.974761

**Published:** 2022-08-09

**Authors:** Benhui Zhao, Yueqing Deng, Lei Cao, Jichun Zhu, Zhengji Zhou

**Affiliations:** ^1^ Miami College of Henan University, Kaifeng, China; ^2^ Key Lab for Special Functional Materials, Ministry of Education, National and Local Joint Engineering Research Center for High-Efficiency Display and Lighting Technology, and School of Materials, Henan University, Kaifeng, China

**Keywords:** CZTSSe, deep defect, open-circuit voltage, solar cells, co-selenization

## Abstract

Kesterite-structured Cu_2_ZnSn(S,Se)_4_ (CZTSSe) thin film photovoltaics have attracted considerable attention in recent years because of its low-cost and eco-friendly raw material, as well as high theoretical conversion efficiency. However, its photovoltaic performance is hindered by large open-circuit voltage (*V*_*OC*_) deficiency due to the presence of intrinsic defects and defect clusters in the bulk of CZTSSe absorber films. The doping of extrinsic cation to the CZTSSe matrix was adopted as an effective strategy to ameliorate defect properties of the solar cell absorbers. Herein, a novel Se&Sb_2_Se_3_ co-selenization process was employed to introduce Sb into CZTSSe crystal lattice. The results reveal that Sb-doping plays an active role in the crystallization and grain growth of CZTSSe absorber layer. More importantly, one of the most seriously detrimental Sn_Zn_ deep defect is effectively passivated, resulting in significantly reduced deep-level traps and band-tail states compared to Sb free devices. As a result, the power conversion efficiency of CZTSSe solar cell is increased significantly from 9.17% to 11.75%, with a *V*_*OC*_ especially enlarged to 505 mV from 449 mV. This insight provides a deeper understanding for engineering the harmful Sn-related deep defects for future high-efficiency CZTSSe photovoltaic devices.

## Graphical Abstract

The open-circuit voltage of Cu2ZnSn(S,Se)4 solar cells increases sharply from 449 to 505 mV due to the passivation of SnZn deep defects by a novel Se&Sb2Se3 co-selenization process.
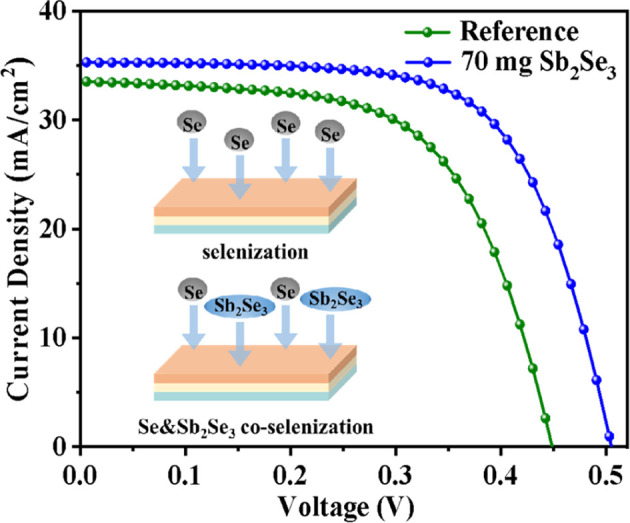

## Introduction

Kesterite Cu_2_ZnSn(S,Se)_4_ (CZTSSe) has been considered as an ideal photovoltaic material after monocrystalline silicon due to its stable structure, benign and earth-abundance raw materials, tunable direct band gap of 1–1.5 eV with a nearly optimal match to the solar spectrum, as well as high optical absorption coefficient (10^4^ cm^−1^) (Wang et al., 2014; Yan et al., 2018; Le Donne et al., 2019; Wang et al., 2022). So far, the highest certified photoelectric conversion efficiency of CZTSSe solar cell has reached 13% (NREL. Best, 2021), which is far from the theoretical conversion efficiency of 32.8% (Shockley and Queisser, 1961). (Shockley-Queisser limit) and its counterpart of CuInGa(S,Se)_2_ (CIGSSe) that has already achieved record efficiency of 23.35% (Nakamura et al., 2019). Among the parameters which affecting the performance of photovoltaic devices, the short-circuit current density (*J*_*SC*_) of high-efficiency CZTSSe solar cells has reached 84% of the theoretical limit, however, the open-circuit voltage (*V*_*OC*_) is only 64% of this value. The large *V*_*OC*_ deficit has become a key bottleneck for the further improving efficiency of CZTSSe solar cells (Su et al., 2020; Du et al., 2021; Gong et al., 2021; Sun et al., 2021; Li et al., 2022).

As a multinary inorganic compound, the crystal structure of CZTSSe is evolved from ZnS, but the stable region of CZTSSe phase is very narrow, which is prone to cause atomic deletion or interatomic displacement in the CZTSSe lattice, forming a variety of defects and defect clusters (Chen et al., 2013; Xu et al., 2021a). The recombination at these intrinsic defects is one of the main factors responsible for its voltage losses. Among the various defects in the bulk absorber, the antisite defect of Cu_Zn_ and Sn_Zn_ is a matter of particular concern in this regard. It was recently demonstrated that the defect clusters of [2Cu_Zn_+ Sn_Zn_] was the origin of band tails (Reya et al., 2017; Ma et al., 2019) and the Sn_Zn_ defect is the main deep trap states in CZTSSe absorber (Biswas et al., 2010; Li et al., 2019). Both of them contribute greatly to the deficit of *V*_*OC*._ Therefore, it is a key strategy to manipulate the Sn-related defects in order to reduce *V*_*OC*_ loss (Kim et al., 2020; Chen et al., 2021).

The Sn_Zn_ defects and [2Cu_Zn_+ Sn_Zn_] defect clusters can be largely suppressed in Sn-poor CZTSSe, however, the Sn-poor composition resulted in poor absorber quality and high concentrations of another detrimental deep defects of Cu_Sn_ (Haass et al., 2018; Xu et al., 2020; Guo et al., 2022), which is also harmful to the solar cell performance. The substitution of Sn with the same group element of Ge is another adoptable measure to mitigate the unfavorable deep defects associated with Sn (Neuschitzer et al., 2018; Xu et al., 2021b). Our recent work has demonstrated that Sn_Zn_ deep traps in CZTSSe absorber layer could be modified by incorporation of small amounts of Ge (Deng et al., 2021), whereas high Ge amounts not only resulted in imperfections of absorber quality because of uncontrolled evaporation of volatile Ge-Se species, but also brought about high density of deep midgap defects, both of which are detrimental to the solar cell performance (Collord and Hillhouse, 2016; Giraldo et al., 2018).

The recent theoretical and experimental results indicate that the Sb dopant in CZTSSe absorber has positive effect on Sn disorder (Zhang et al., 2017; Tiwari et al., 2018), which can been rationalized by their small difference in ionic radii, resulting in smaller lattice relaxations and lower formation energy of Sb_Sn_ antisites compared with other intrinsic Sn-related defects in CZTSSe. In this work, Sb^3+^ was introduced into CZTSSe absorber by a modified selenization process using both of Sb_2_Se_3_ and Se as evaporation source. The effect of different amount of Sb_2_Se_3_ source on the morphology, element distribution of CZTSSe absorber, as well as photovoltaic performance of final CZTSSe solar cells were systematically studied. Furthermore, using deep-level transient spectroscopy (DLTS), the conclusive evidence that Sb^3+^ doping could suppress a number of harmful Sn_Zn_ defect states within CZTSSe was provided. Benefitting from the engineering of the defect characteristics via Sb^3+^ doping, the *V*_*OC*_ of CZTSSe solar cell is enhanced from 449 to 505 mV, achieving a champion efficiency of 11.75%.

## Materials and methods

### Materials

Nano powder of copper (Cu, 99.9%) was purchased from Macklin Reagent Company (China). Zinc powder (Zn, 99.9%), tin powder (Sn, 99%), sulfur powder (S, 99.9%), selenium powder (Se, 99.9%), antimony triselenide pellets (Sb_2_Se_3_, 99.99%), thiourea (NH_2_CSNH_2_, 99%); cadmium sulfate (CdSO_4_·8/3H_2_O, 99%), ethanolamine (C_2_H_7_NO, 99.5%), and 2-methoxyethanol (C_3_H_8_O_2_, 99.7%) were purchased from Aladdin. Ammonium hydroxide (NH_4_OH, 25%) was purchased from Tianjin Fuyu Fine Chemical Co., Ltd. Thioglycolic acid (C_2_H_4_O_2_S, 98%) was purchased from Acros Organics Company. 1,2-ethylenediamine (H_2_NCH_2_CH_2_NH_2_, 99%) and 2-ethanedithiol (HSCH_2_CH_2_SH, 98+%) were obtained from Alfa Aesar. All chemicals used in this study were used without further purification.

### Fabrication of kesterite absorber film and solar cells

CZTSSe precursor solution was prepared by dissolving the elemental Cu, Zn, Sn, S, and Se into the mixture of 1,2-ethanedithiol and 1,2-ethylenediamine, which was described by the authors’ previous works (Deng et al., 2021), CZTSSe precursor films were obtained by spin-coating the precursor solution on Mo substrate, followed by sintering on a hot plate at 330°C for 3 min. This spin-coating and annealing procedure was repeated several times in a glove box until a desired thickness of 2 µm was obtained. The normal selenization processes were conducted in a rapid thermal processing (RTP) furnace using a graphite box containing the as-prepared CZTSSe films and excess selenium particles at 550°C for 15 min. For the co-selenization process, extra Sb_2_Se_3_ with mass of 60, 70 and 80 mg respectively was placed into the identical graphite box before annealing. After selenization, a buffer layer of CdS, as well as window layer of ZnO and ITO were successively deposited onto the CZTSSe thin films by chemical bath deposition and magnetron sputtering. The CZTSSe solar cells were finally finished with thermal evaporated Ag top grid contact.

### CZTSSe film and device characterization

X-ray diffraction (XRD) measurements were carried out using a Bruker AXS (D8 Advance) with Cu Ka radiation (1.5405 Å). Raman spectra were measured with a Renishaw in Via Raman microscope system with a 532 nm wavelength excitation laser. The morphology and compositional analysis were performed by a field emission scanning electron microscope equipped with Energy Dispersive X-ray spectroscopy (EDS) (FESEM, Nova Nano SEM 450). The J–V curves and parameter were tested with a solar simulator (Zolix SS150) with an AM1.5 solar spectrum filter. EQE was carried out using Zolix QE system (SCS100). The DLTS measurements were carried out using an FT-1030 HERA DLTS system equipped with a JANIS VPF-800 heat controller. Electrochemistry impedance spectroscopy (EIS) were measured using an Autolab electrochemical workstation (AUT302N).

## Results and discussion

**FIGURE 1:**
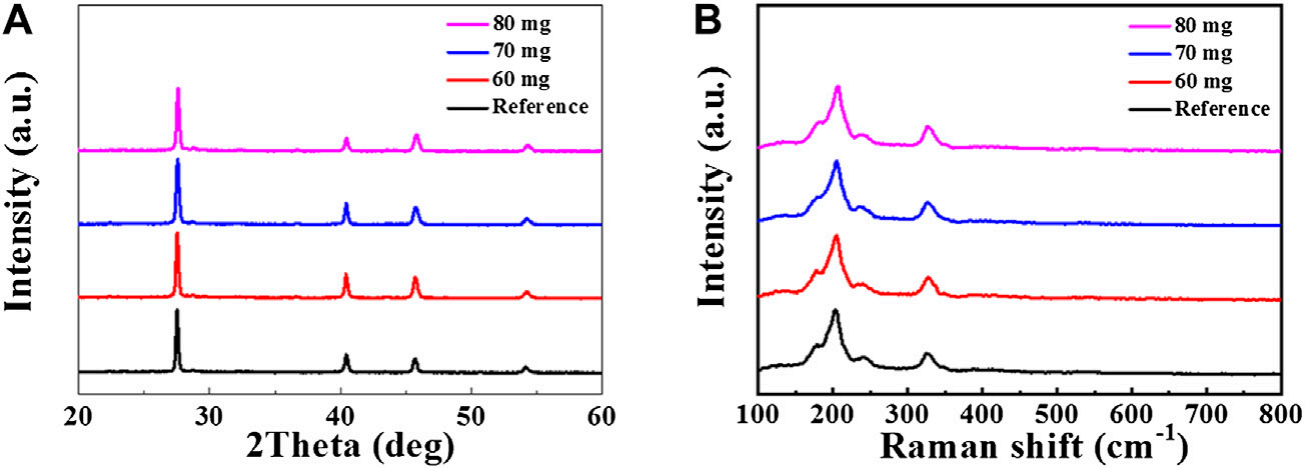
**(A)** XRD pattern and **(B)** Raman spectra of CZTSSe films selenized with different Sb_2_Se_3_ contents.

In order to introduce Sb into the CZTSSe absorption layer, Sb_2_Se_3_ & Se mixed atmosphere was employed in the selenization of CZTSSe precursor film. To identify the change in the crystal structure of annealed CZTSSe films, X-ray diffraction (XRD) patterns of the selenized CZTSSe thin films with different Sb_2_Se_3_ content ranging from 60 to 80 mg were measured. At the same time, the customary selenizing films using only Se source was also examined as reference. As shown in Figure 1A, all samples exhibit a pure kesterite crystal structure with main diffraction peaks of 27.28°, 45.25°, and 53.64°, which can be indexed to the (112), (204), and (312) planes of the CZTSSe phase (JCPDS # 52-0868) (Xiao et al., 2016; Li et al., 2021). It is clear that introduction of Sb_2_Se_3_ in selenization did not bring additional secondary phase. To further confirm the phase purity of selenized CZTSSe thin films, Raman spectra were carried out and shown in Figure 1B. The samples with different selenization condition present almost similar Raman spectra. All the peaks in the selenized thin films were consistent well with kesterite structure of the CZTSSe. No distinct peaks corresponding to any binary and ternary impurity phases could been detected. Thus, according to the XRD and Raman spectra results, it is reasonable to conclude that Sb-doped CZTSSe thin films without any possible impurity phases were obtained in the co-selenization annealing treatment with Se and Sb_2_Se_3_.

**FIGURE 2:**
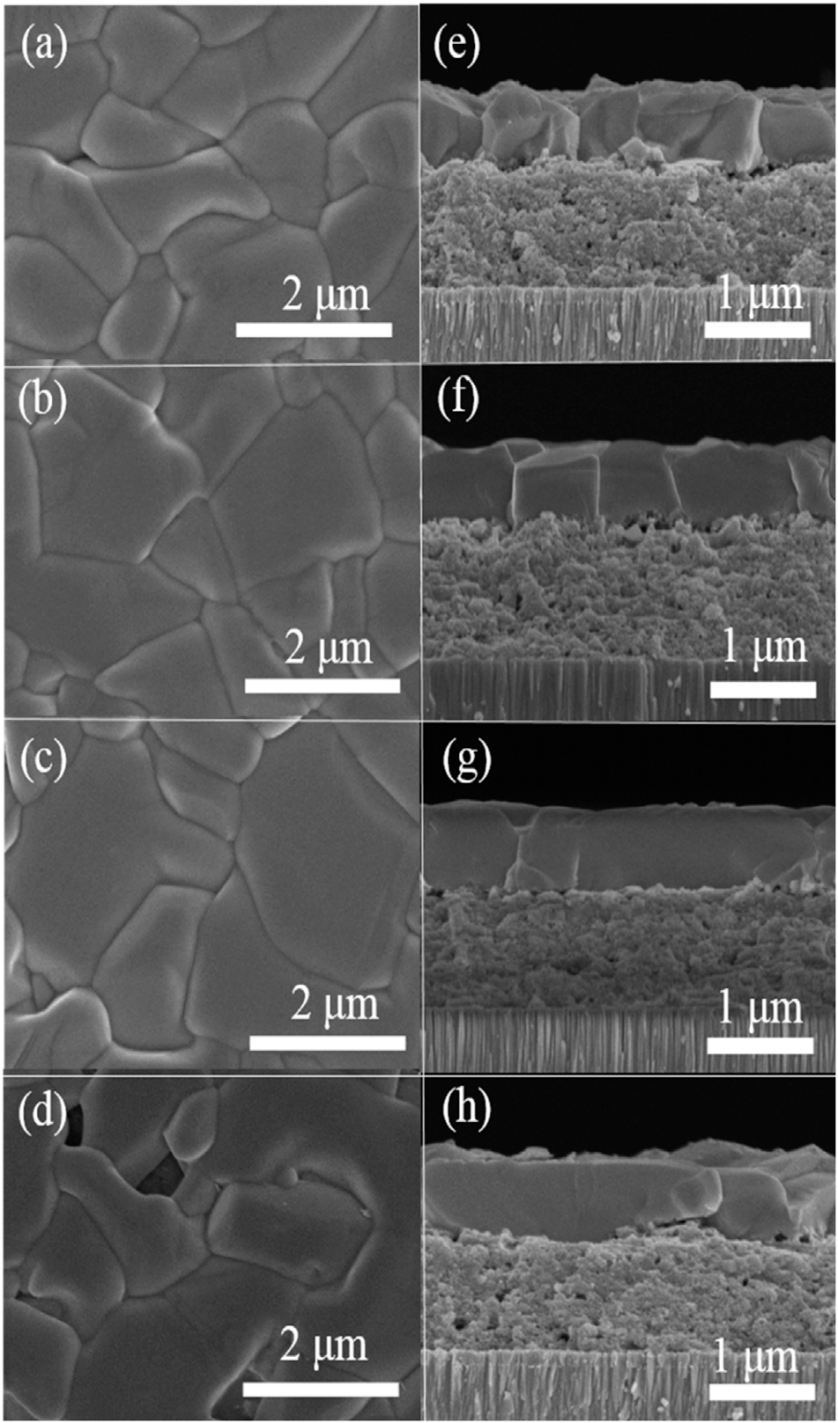
Top-view and cross sectional SEM images of CZTSSe films selenized with different Sb_2_Se_3_ contents: **(A)** and **(E)** 0 mg, **(B)** and **(F)** 60 mg, **(C)** and **(G)** 70 mg, **(D)** and **(H)** 80 mg.

To investigate the effect of Sb-doping on the microscopic morphology of the selenized CZTSSe film, scanning electron microscopy (SEM) characterization on samples annealed under different quantity of Sb_2_Se_3_ were performed. As shown in Figure 2, the top surface of CZTSSe films are composed of large grains in a few micrometers after selenization. When the CZTSSe precursor film was annealed under a Se and Sb_2_Se_3_ environment, the SEM images (Figures 2B,C) display a clear increase in the grain sizes, indicating that the Se&Sb_2_Se_3_ co-selenization process can significantly promote the growth of grains. However, a few voids can be seen on the surface of CZTSSe films when the quantity of Sb_2_Se_3_ was further increased to 80 mg (Figure 2D), which can been ascribed to the overgrowth of CZTSSe films induced by incorporation of Sb (Liu et al., 2021). On the basis of the previous results, the accelerative grain growth of kesterite thin films by Sb doping was resulted from the liquid-assisted grain growth due to the formation of low-melting Sb_2_Se_3_ during the annealing process (Guo et al., 2015; Cai et al., 2020). The cross-sectional SEM images indicate that the CZTSSe films are composed by a typical double-layer structure with bigger grains at the surface and smaller grains at the bottom. In addition, the incorporation of Sb resulting in increasing the grain size at the surface can be further demonstrated from the cross section SEM images. The bigger crystals reduce the grain boundaries at the top CZTSSe layer, accordingly reducing the recombination of photo-generated carriers, which is beneficial to the *V*_*OC*_ of a CZTSSe photovoltaic device. The EDS mapping images of Sb-doped CZTSSe films revealed that Sb is evenly distributed in the entire absorption layer, consistent with the distribution of other metal elements of Cu, Zn and Sn (Supplementary Figure S1), indicating that there is no obvious phase separation in the Se&Sb_2_Se_3_ co-selenized CZTSSe films, which is in good agreement with the XRD and Raman results.

**FIGURE 3:**
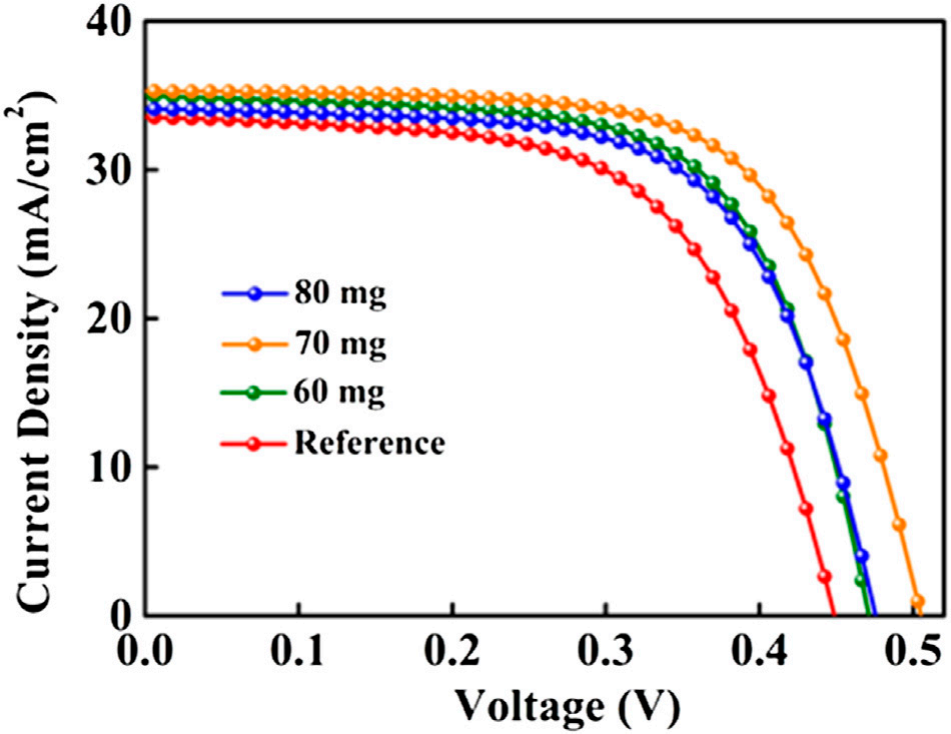
J-V curves of the best devices with CZTSSe films selenized under different Sb_2_Se_3_ contents.

**FIGURE 4:**
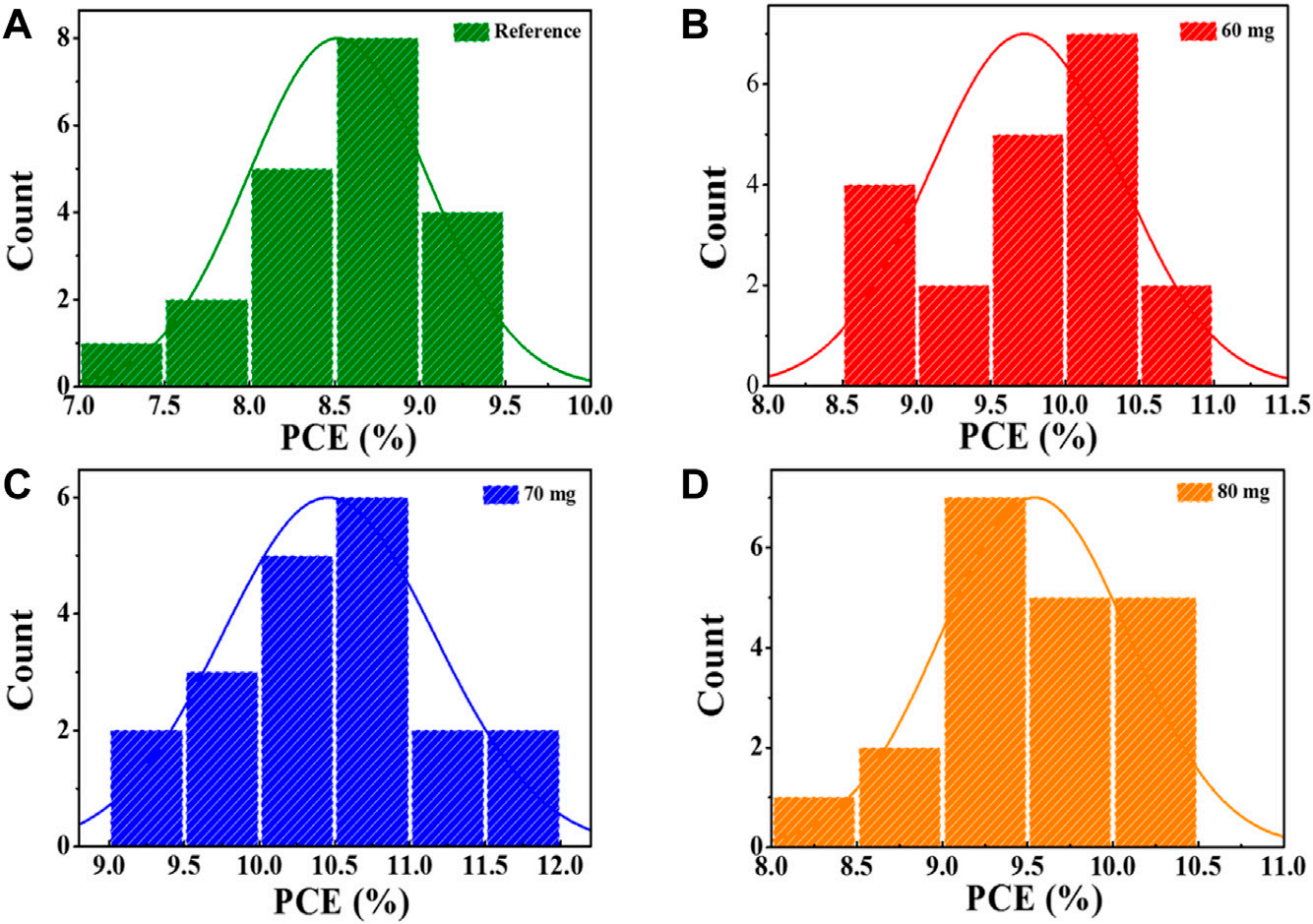
Statistical distribution of solar cell efficiency with CZTSSe films selenized under different Sb_2_Se_3_ contents: **(A)** 0 mg, **(B)** 60 mg, **(C)** 70 mg, **(D)** 80 mg

The influence of Se&Sb_2_Se_3_ co-selenization and incorporation of Sb into CZTSSe absorber layer on final solar cell performance was evaluated. Figure 3 presents the *J-V* characteristics of the best-performing CZTSSe photovoltaic devices with different amount of Sb-doping. The devices used CZTSSe films selenized annealing only in a Se environment exhibited a power conversion efficiency (PCE) of 9.17% with short-circuit current density (*J*_*SC*_), *V*_*OC*_, and FF values of 33.55 mA cm^−2^, 449 mV, and 60.94%, respectively. The CZTSSe films selenized with Se and 70 mg Sb_2_Se_3_ produced the best PCE of 11.75%, with a *V*_*OC*_ of 505 mV, a *J*_*SC*_ of 35.31 mAcm^−2^ and a FF of 65.88%. All the three photovoltaic parameters increase with increasing Sb_2_Se_3_ content using in selenization, especially the *V*_*OC*_. With further increase of the quantity of Sb_2_Se_3_ to 80 mg, the *V*_*OC*_ exhibits an obvious drop, while *J*_*SC*_ and FF show slight decrease, probably because the voids formed at the surface of CZTSSe films have deteriorated the quality of p-n junction, as demonstrated by SEM results. The corresponding photovoltaic parameters extracted from the *J-V* curves are shown in Table 1. Figure 4 shows statistical distributions of PCE recorded from 20 individual cells of each selenization condition, and the mean of photovoltaic parameters are displayed in Supplementary Table S1, which further demonstrated the efficiency of the Sb doped devices is significantly improved. Moreover, the distribution is relatively concentrated, indicating good repeatability and credibility.

**TABLE 1:** Summary of the Photovoltaic Performances for CZTSSe devices with the absorber selenized under different condition.

Device	*V*_*OC*_ (mV)	*J*_*SC*_ (mA/cm^2^)	FF (%)	PCE (%)
References	449	33.55	60.94	9.17
60 mg	471	34.99	65.54	10.81
70 mg	505	35.31	65.88	11.75
80 mg	476	34.10	64.60	10.48

**FIGURE 5:**
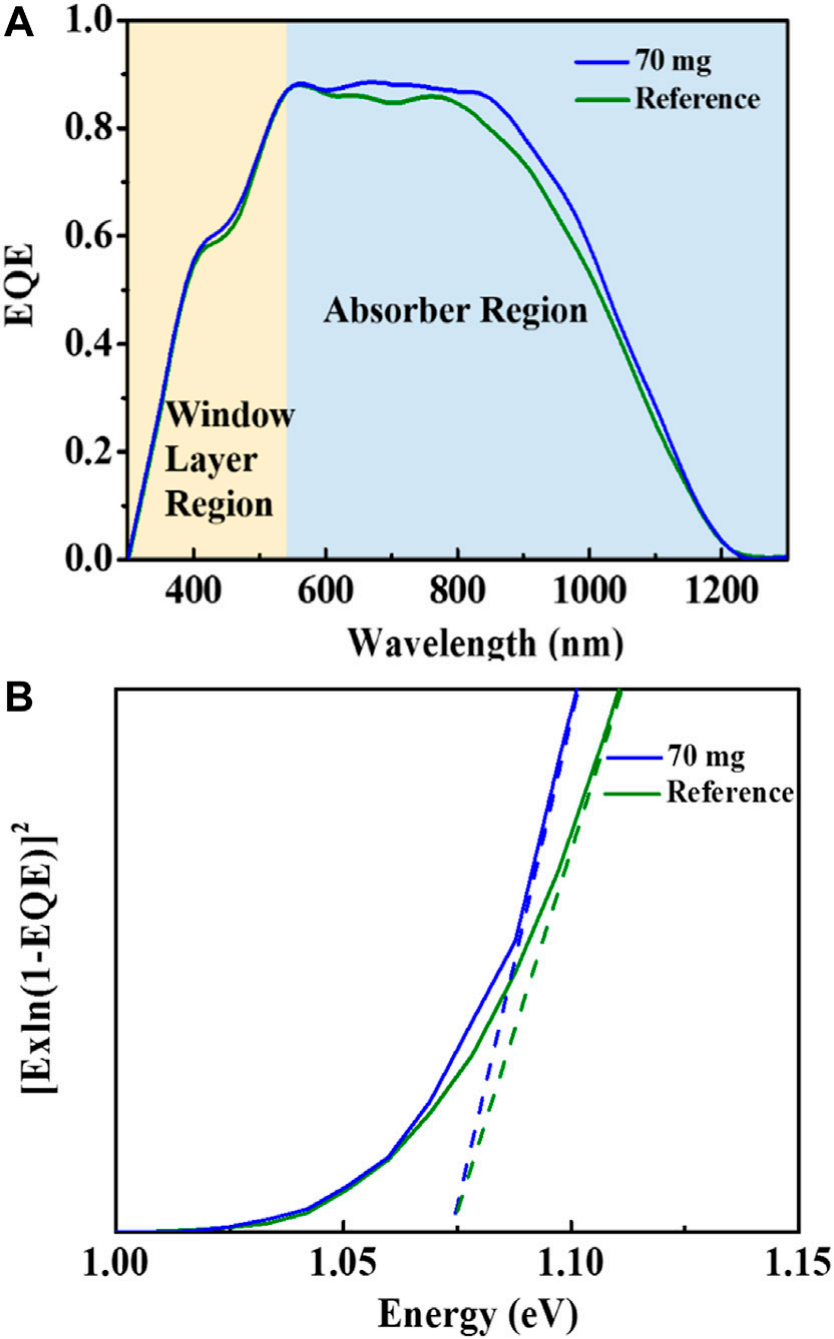
EQE curves **(A)** and bandgap estimation **(B)** of the champion pristine CZTSSe device and Sb doped-CZTSSe device.

The external quantum efficiency (EQE) curves of the best-performing CZTSSe solar cells with and without Sb-doping were compared, as displayed in Figure 5A. It is observed that the EQE are almost the same in the wavelength range of 350-550 nm. However, in the range of 550-1100 nm, the Sb-doped CZTSSe solar cells exhibits a slight increase of light response, indicating better quality of CZTSSe/CdS heterojunction and fewer defect energy level in the absorber layer than the control device. The corresponding bandgaps of CZTSSe films are compared in Figure 5B, which are graphically determined from the EQE data by plotting [E×ln (1−EQE)]^2^ versus E. As can be seen, there is no obvious change in bandgaps of CZTSSe films using a standard selenization process and Se&Sb_2_Se_3_ co-selenization process, which are all calculated as 1.075 eV, suggesting the significant *V*_*OC*_ improvement of the Sb-doped CZTSSe devices was not lies in the bandgap change of CZTSSe absorber layer. In addition, Urbach band-tail analysis was performed to examine the band tailing for both devices. As depicted in the Supplementary Figure S2, the value of Urbach tail energy (E_u_) is estimated to be 52 and 30 meV for the pristine CZTSSe device and Sb doped-CZTSSe device. A distinctly lower Eu for the absorber with Sb-doping indicates that the band tailing is reduced compared with that of the undoped CZTSSe film, which is probably linked to the decreased amount of antisite defects upon Sb incorporation (Zhao et al., 2021).

**FIGURE 6:**
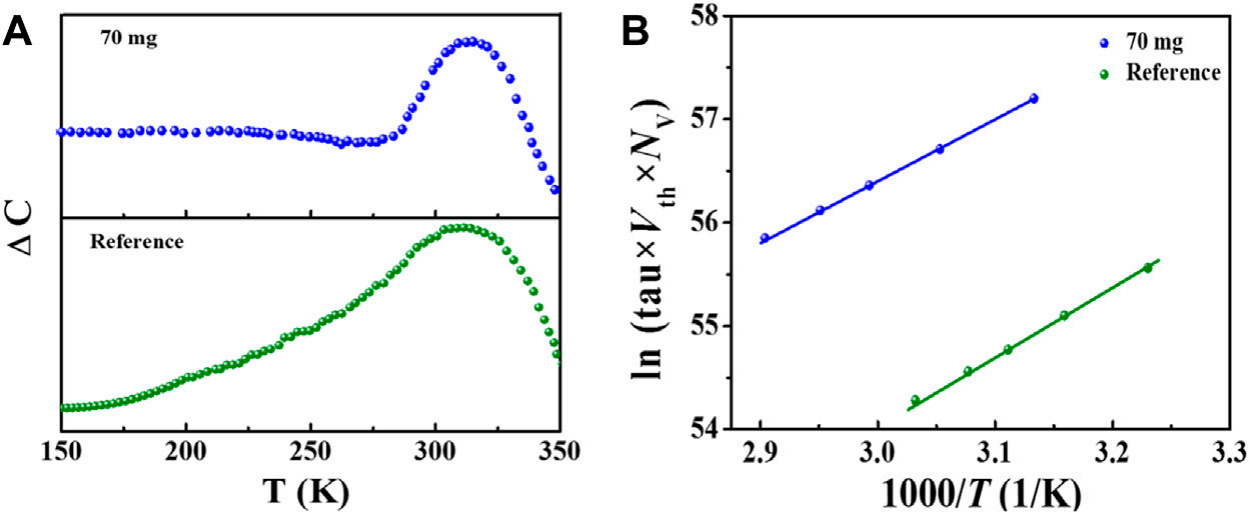
**(A)** DLTS measurements of the champion pristine CZTSSe device and Sb doped-CZTSSe device, **(B)** Arrhenius plots corresponding to the peaks derived from the DLTS spectra.

The underlying mechanism for Sb-doping induced performance improvement is further analyzed by DLTS, aiming at gaining insights on change of defects properties in the Sb-incorporated CZTSSe absorber layer. DLTS measurements were carried out on the best-performing CZTSSe devices with and without incorporation of Sb. Figure 6 shows the results of capacitance-mode DLTS (C-DLTS) measurement at the temperature range of 150–280 K, and the reverse biased (V_R_), pulse voltage (V_P_), and pulse width were set at 0.4 V, - 0.2 V, and 10 ms, respectively. As can be seen from Figure 6A, both devices display an obvious peak at about 310K, indicating that a deep level defect is detected here. Compared with the reference device, the DLTS peak of the CZTSSe device treated with 70 mg Sb_2_Se_3_ is significantly narrowed, demonstrating a faster carrier emission rate and thus a more minor carrier recombination. The defect activation energies (E_a_) and density (N_T_) deduced from the Arrhenius curves of ln (τV_th_N_v_) versus 1000/T are depicted in Figure 6B, and the detailed values are shown in Table 2. According to the reported value of E_a_ in previous literatures (Nisika et al., 2020), the measured deep-level defects with E_a_ of 0.558 eV for pristine and 0.507 eV for Sb-doped CZTSSe device can been identified as Sn_Zn_ donor defects. One can see that the incorporation of Sb significantly reduced the E_a_ of Sn_Zn_ donor defects, which could speed up the emission rate of minority carriers from the electron trapping. Furthermore, over one-order-lower defect density upon Sb introduction has been demonstrated as expected. In prior research, it has been verified that Sn_Zn_ antisite defect was the main reason for large *V*_*OC*_ loss (Li et al., 2019; Du et al., 2021). Therefore, the sharp improved *V*_*OC*_ in Sb-doped kesterite photovoltaic devices can been well explained by the suppression of Sn_Zn_ deep-donor states in CZTSSe absorber layer.

**TABLE 2:** Sn_Zn_ deep defect metrics of the champion pristine CZTSSe device and Sb doped-CZTSSe device.

Device	Peak temp (K)	*E*_a_ (eV)	*N*_T_ (cm^−3^)	Defect level
References	310	0.558	9.91×10^13^	Sn_Zn_
70 mg	307	0.507	3.80×10^12^	Sn_Zn_

**FIGURE 7:**
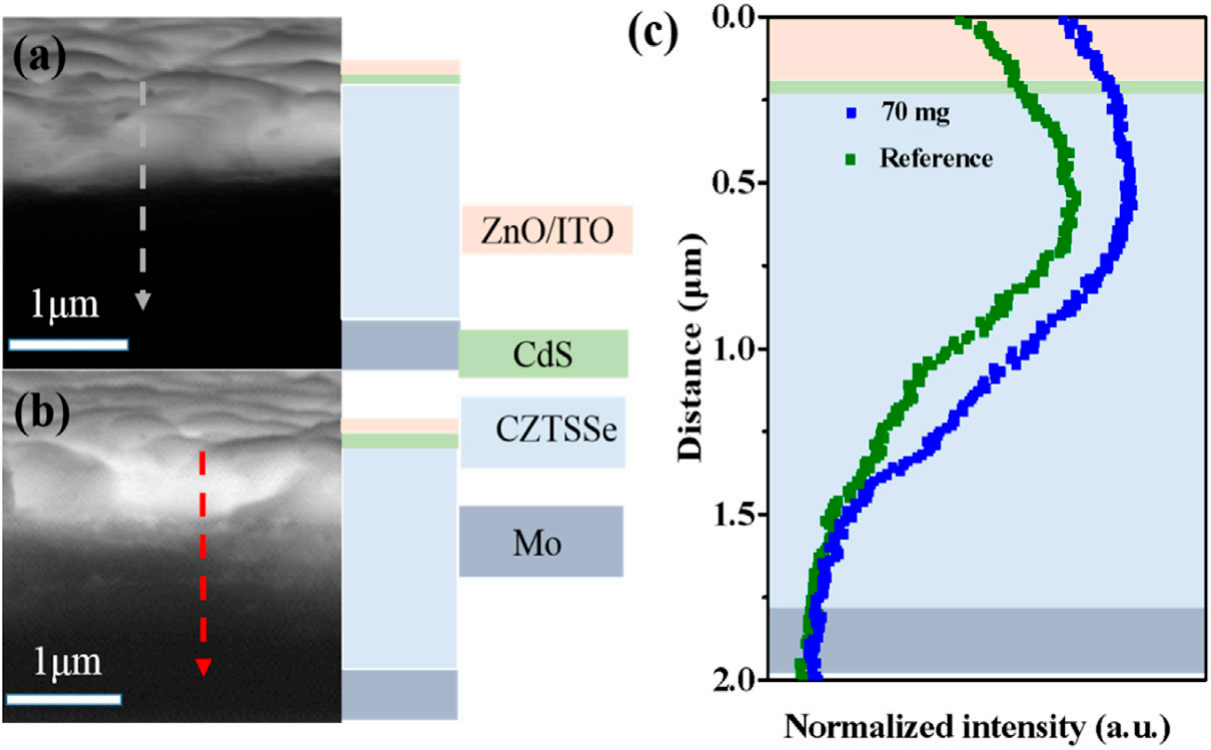
The EBIC images of pristine CZTSSe device **(A)** and Sb doped-CZTSSe device **(B)**, **(C)** is the corresponding normalized intensity profiling along the dashed line in **(A,B)**.

Finally, to further illustrate the effect of Sb doping on the collection efficiency of the CZTSSe photovoltaic devices, electron beam-induced current (EBIC) measurements were performed on the pristine and 70 mg Sb_2_Se_3_ treated devices. Figures 7A,B comparatively show the EBIC images across the cross section of the two devices. Bright areas in the EBIC image indicate regions of effective collection of minority carriers, and its depth is a reflection of depletion width and minority diffusion length (Chantana et al., 2019; Raghuwanshi et al., 2020). As can be seen that the bright regions are mainly located at the upper absorber layer, indicating high-efficiency collection of photo-generated carriers at the top of large-grained CZTSSe films. From the normalized EBIC signals along the dotted arrow of both CZTSSe devices (Figure 7C), it is clear that the EBIC signal is dramatically enhanced after Sb incorporation in the regions from CZTSSe/CdS p-n junction to upper CZTSSe film, implying the improved carrier collection capability and prolonged minority diffusion length, which can be ascribed to substantially reducing Sn_Zn_ deep defects. The EBIC findings echo the DLTS results above and clearly demonstrate that Sb doping is beneficial to quenching the Sn_Zn_ deep-trap levels in CZTSSe absorber layer and hence reduce nonradiative recombination in CZTSSe photovoltaic devices. EIS was further applied to probe the characteristics of minority carrier recombination, as shown in Supplementary Figure S3. A significantly enhanced recombination resistance is observed in CZTSSe solar cells treated with 70 mg Sb_2_Se_3_, indicating Sb-doping can decrease the recombination center in the bulk of CZTSSe film, which is consistent with the EBIC results.

## Conclusion

In summary, this work presents an effective approach to introduce Sb into CZTSSe absorber layer by a facile Sb_2_Se_3_ and Se co-selenization process. The influence of Sb_2_Se_3_ contents used during the co-selenization process on the morphology and structure, as well as electrical properties of CZTSSe films was evaluated. With the optimized doped content of Sb, the CZTSSe solar cell efficiency enhanced from 9.17% to 11.75%, with an open-circuit voltage progressively increased to 505 from 449 mV. DLTS and EBIC results revealed that Sn_Zn_ antisite defects were dramatically passivated because of the incorporation of Sb, resulting in significantly reduced deep trap density and improved collection ability. This study provides a simple and promising doping strategies for engineering the defect characteristics in kesterite film, which would help decrease the *V*_*OC*_ deficit for future high-performance kesterite solar cells.

## Data Availability

The original contributions presented in the study are included in the article/Supplementary Material, further inquiries can be directed to the corresponding authors.
