# Supplementary material for: Doping of Sb into Cu2ZnSn(S,Se)4 absorber layer via Se&Sb2Se3 co-selenization strategy for enhancing open-circuit voltage of kesterite solar cells
Source: Front Chem. 2022 Aug 9;10:974761. doi: 10.3389/fchem.2022.974761 (PMC9395640; doi:10.3389/fchem.2022.974761)
Supplement: Supplementary file 1 [file DataSheet1.docx]

**Supporting Information**

**Doping of Sb into Cu_2_ZnSn(S,Se)_4_ absorber layer via Se&Sb_2_Se_3_ co-selenization strategy for enhancing open-circuit voltage of kesterite solar cells**

Benhui Zhao^a^, Yueqing Deng^b^, Lei Cao^b^, Jichun Zhu^a*^, Zhengji Zhou^b*^

^a^ *Miami College of Henan University, Kaifeng 475004, China*

^b^ *Key Lab for Special Functional Materials, Ministry of Education, National and Local Joint Engineering Research Center for High-Efficiency Display and Lighting Technology, and School of Materials, Henan University*

^*^Corresponding author.

*E-mail address:* zjc@henu.edu.cn (J. Zhu), zzj@henu.edu.cn (Z. Zhou),


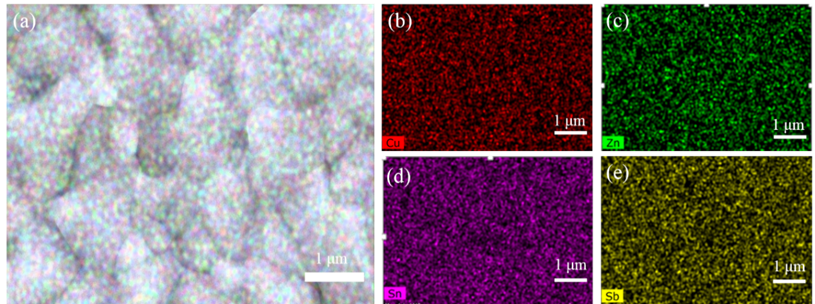


**Figure S1.** EDS mapping images for Cu, Zn, Sn and Sb elements in the CZTSSe film selenized with additional 70 mg Sb_2_Se_3_.

**Table S1.** Statistical performance parameters for CZTSSe devices with the absorber selenized under different condition.

| Device | *V_OC_*  (mV) | *J_SC_*  (mA/cm^2^) | FF  (%) | PCE  (%) |
| --- | --- | --- | --- | --- |
| Reference | 443 | 32.10 | 60.08 | 8.54 |
| 60 mg | 466 | 33.84 | 63.75 | 10.06 |
| 70 mg | 492 | 33.05 | 64.51 | 10.50 |
| 80 mg | 462 | 33.92 | 63.23 | 9.90 |


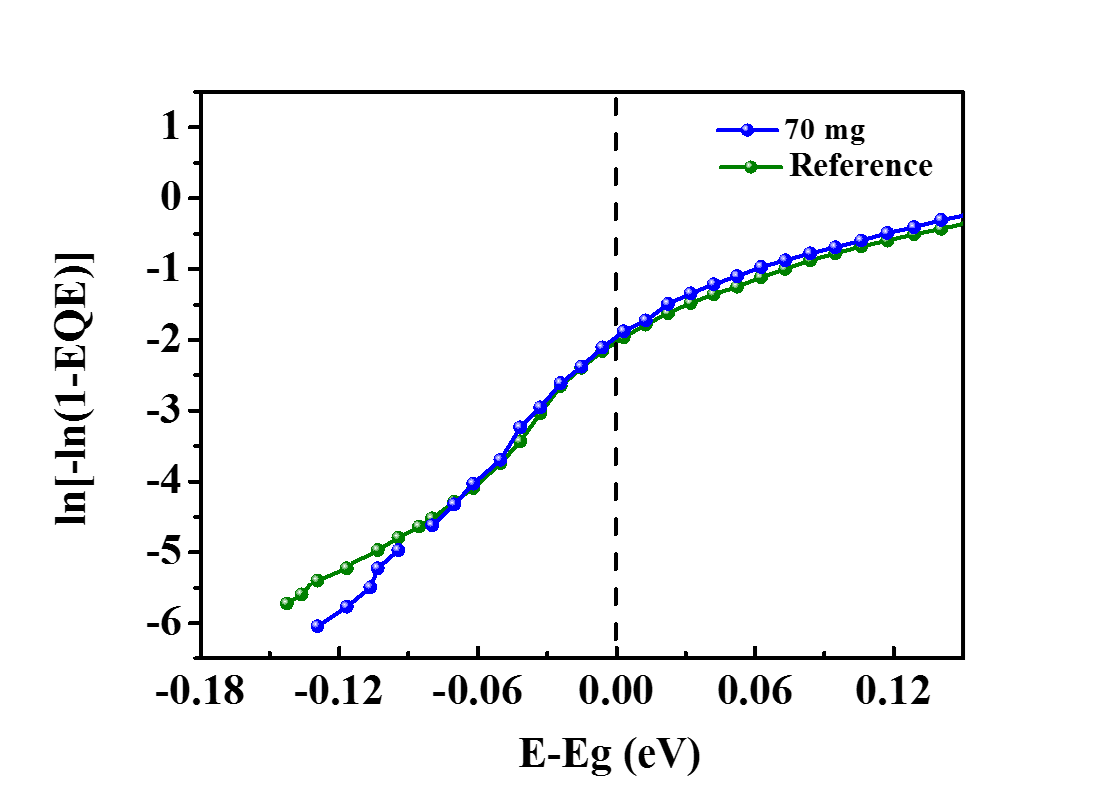


**Figure S2.** Urbach tail analysis for the champion pristine CZTSSe device and Sb doped-CZTSSe device.


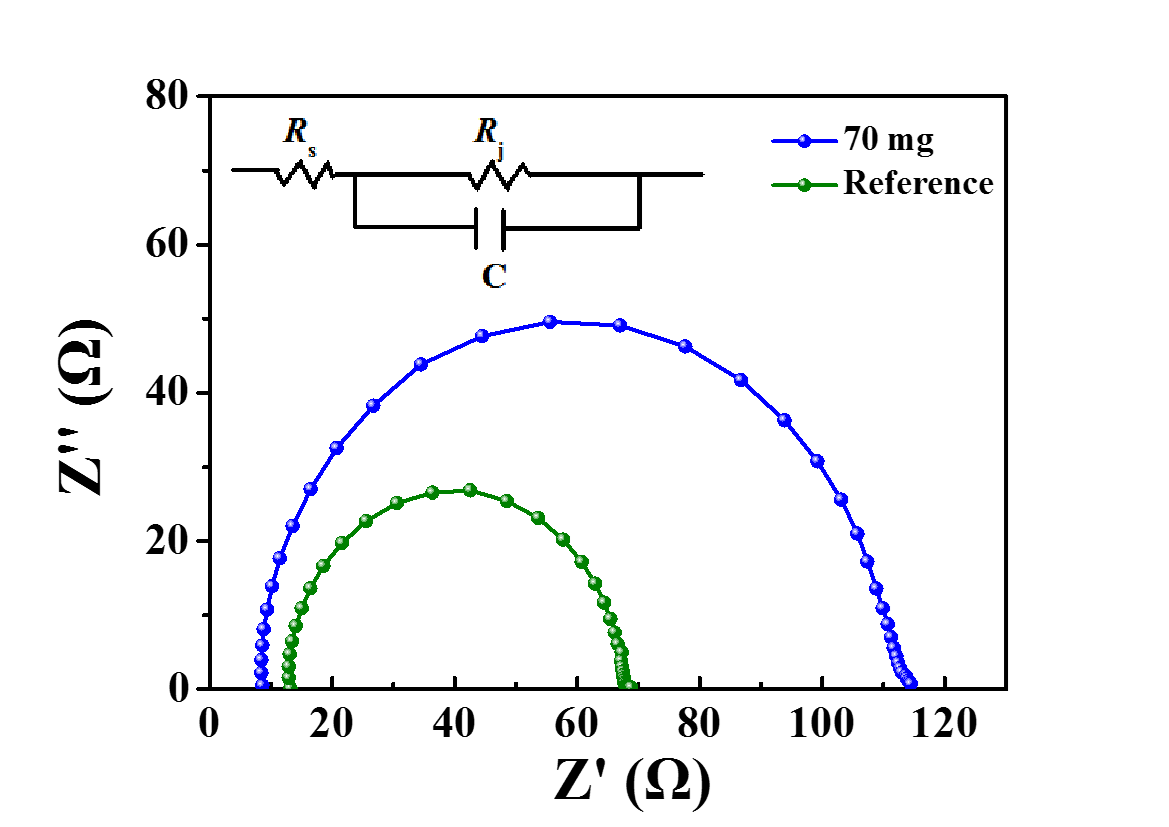


**Figure S3.** EIS Nyquist plots of the champion pristine CZTSSe device and Sb doped-CZTSSe device.
